# Development of PET insert for simultaneous PET/MR imaging of human brain

**DOI:** 10.1186/2197-7364-1-S1-A8

**Published:** 2014-07-29

**Authors:** Jiwoong Jung, Yong Choi, Jin Ho Jung, Sangsu Kim, Ki Chun Im, Hyun Keong Lim, Changheun Oh, HyunWook Park, Gyuseong Cho

**Affiliations:** Molecular Imaging Research & Education (MiRe) Laboratory, Department of Electronic Engineering, Sogang University, Seoul, Korea; Departments of Electrical Engineering and Nuclear and Quantum Engineering, Korea Advanced Institute of Science and Technology (KAIST), Daejeon, Korea

Recently, there has been great interest on the development of combined PET/MR, which is a useful tool for both functional and anatomic imaging. The purpose of this study was to develop a MR compatible PET insert for simultaneous PET and MR imaging of human brain and to evaluate the performance of the hybrid PET-MRI. The PET insert consisted of 18 detector blocks arranged in a ring of 390 mm diameter with 60 mm axial FOV. Each detector block was composed of 4 × 4 matrix of detector modules, each of which consisted of a 4 × 4 array LYSO coupled to a 4 × 4 GAPD array. The PET gantry was shielded with gold-plated conductive fabric tapes. The charge signals of PET detector transferred via 4 m long flat cables were fed into the position decoder circuits (PDCs) and then transferred to FPGA-embedded DAQ modules. The PDCs and DAQ modules were enclosed in an aluminum box and located at the rear of the MR bore inside MRI room. 3-T human MRIs of two different vendors were used to evaluate the MR compatibility of developed PET insert. No significant changes of the PET performance and the homogeneity of MR images caused by the non-compatibility of PET-MRI were observed with the 2 different MRIs. The signal intensities of MR images were slightly degraded (<3.6%) with the both MRI systems. The difference between independently and simultaneously acquired PET images of brain phantom was negligibly small (<4.3%). High quality simultaneous brain PET and MRI of 3 normal volunteers were successfully acquired. Experimental results indicate that the high performance compact and lightweight PET insert for hybrid PET/MRI, which could be utilized with the MRI from various manufactures, can be developed using GAPD arrays and charge signal transmission method proposed in this study.

